# Interposer-Based ESD Protection: A Potential Solution for μ-Packaging Reliability of 3D Chips

**DOI:** 10.3390/mi16040488

**Published:** 2025-04-21

**Authors:** Xunyu Li, Zijin Pan, Weiquan Hao, Runyu Miao, Zijian Yue, Albert Wang

**Affiliations:** Department of Electrical and Computer Engineering, University of California, Riverside, CA 92521, USA

**Keywords:** AI, CDM, chip, ESD protection, heterogeneous integration, IC, interposer, reliability, SoIC, μ-packaging

## Abstract

The ending of Moore’s Law calls for innovations in integrated circuit (IC) technologies and chip designs. Heterogeneous integration (HI) emerges as a pathway towards smart future chips for more Moore time and for beyond-Moore time, featuring systems-on-integrated-chiplets (SoICs) and advanced micro-packaging (μ-packaging). Reliability, particularly with regard to electrostatic charge (ESD) failure, is a major challenge for 3D SoIC chips in μ-packaging, which is an emerging design-for-reliability challenge for future chips. This perspective article articulates that interposer-based ESD protection will be an important potential solution for 3D SoIC chips in μ-packaging against the devastating ESD failure problem.

## 1. Motivation

**Figure 1:**
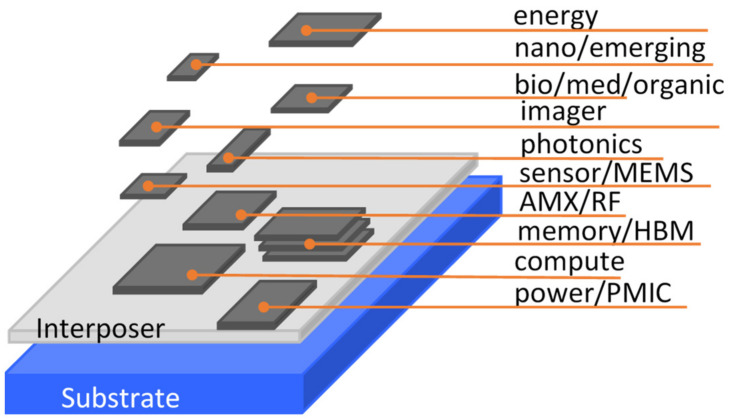
Conceptual illustration of HI-enabled chiplet-based SoIC with rich functionalities and featuring an interposer.

Semiconductors and integrated circuits (ICs) have forever changed human life, with microelectronics chips penetrating into every sector of modern society. Since the invention of transistors and ICs [1,2,3,4], Si complementary metal-oxide-semiconductor (CMOS) technologies have been relentlessly advancing at a pace driven by Moore’s Law [5]. Nevertheless, simple CMOS scaling is rapidly approaching its physical limitation and the ending of Moore’s Law urgently calls for disruptive innovations in IC technologies in order to sustain the continuous advances in microelectronics needed to meet the demand for the higher performance of chips. Fortunately, heterogeneous integration (HI) has emerged and opened a pathway towards smart future chips that can deliver not only extreme performance, but also rich functionalities, while ensuring affordability [6]. In a nutshell, HI technologies hetero-integrate different devices made in dissimilar materials using different technologies into a superchip of higher performance and more functionalities, mainly leveraging CMOS IC process platforms. These devices of different functions are broadly called chiplets (generally on the same materials platform) or dielets (typically when using different materials), which can be put together into systems-on-integrated-chiplets (SoICs), as depicted in Figure 1. The IC-scale integration makes SoICs comparable to monolithic systems-on-chip (SoC) in terms of performance, footprint and costs. Critically, reliability and packaging are the two key factors that vitally affect HI/chiplet-based SoIC chips [7].

Advanced packaging plays a key role in realizing HI-enabled SoIC chips where materials and processing innovations are required to address important attributes such as performance (e.g., chiplet-to-chiplet data bandwidth), thermal management (e.g., cooling), integrability, manufacturability, yield and costs, etc. Uniquely, CMOS fabrication technologies can be used to achieve micro-packaging (μ-packaging), realizing fine-pitch critical dimensions on packaging substrates (or any carrier media) down to the μm to nm level, in order to make SoICs similar to SoCs in terms of both performance and footprint, something which is entirely different from traditional packaging technologies at board level, e.g., for conventional systems-in-packaging (SiP). As such, advanced 3D μ-packaging emerges as a vital technology in modern chip design and fabrication today, with, for example, the highly successful chip-on-wafer-on-substrate CoWoS packaging technology [8,9]. Key technical features enabling μ-packaging include 2D/2.xD/3D packaging, various packaging substrates (e.g., Si, glass, organic), ball bonding, through vias (e.g., through-silicon via, aka, TSV), passive and active interposers, embedded bridges (e.g., Si bridge), micro bumps, copper pillars, Cu–Cu bonding, integrated passive devices (IPDs), flip-chip, 3D stacking, ball grid arrays (BGAs), fine-pitch interconnect fabrics, redistribution layers (RDLs), wafer-level packaging (WLP), etc. [6]. Essentially, today’s μ-packaging is quite similar to, and requires concurrent engineering with, IC fabrication.

Reliability is a major challenge for making SoIC chips of high performance and at affordable cost because heterogeneous integration makes reliability much more complicated [10,11]. While many reliability problems exist for HI technologies and microsystems, electrostatic discharge (ESD) is particularly challenging for SoIC chips in advanced μ-packaging [12,13]. What makes ESD reliability extremely complicated and ESD protection designs very challenging is the heterogeneity—the core nature of HI technologies. SoIC chips typically have numerous functional domains, such as, logic and compute (e.g., CPU, GPU, AI engine), analog and mixed-signal (AMX), memories (e.g., high-bandwidth memories, aka, HBM), radio-frequency (RF) wireless, power supplies and power management ICs (PMIC), ultra-low power CMOS, high-voltage high-power (HV/HP), energy conversion (e.g., solar), optoelectronics and photonics (e.g., lasers), sensors and actuators, MEMS/NEMS, organic and bio-medical electronics, imagers (e.g., CMOS/CIS), quantum, neuromorphic computing, and various emerging and nano device functions. Similarly, SoICs/μ-packaging involves various different materials (from Si to compound semiconductors, ultra/wide-band gap semiconductors, low-dimensional and nano materials, organic, etc.), complex chip architectures, and complicated physical boundaries. Together, heterogeneous integration and SoIC not only makes ESD protection design more difficult, but also often leads to unexpected new ESD phenomena.

## 2. Interposer-Based ESD Protection for Chips in μ-Packaging

### 2.1. ESD Protection and Design Challenges

**Figure 2:**
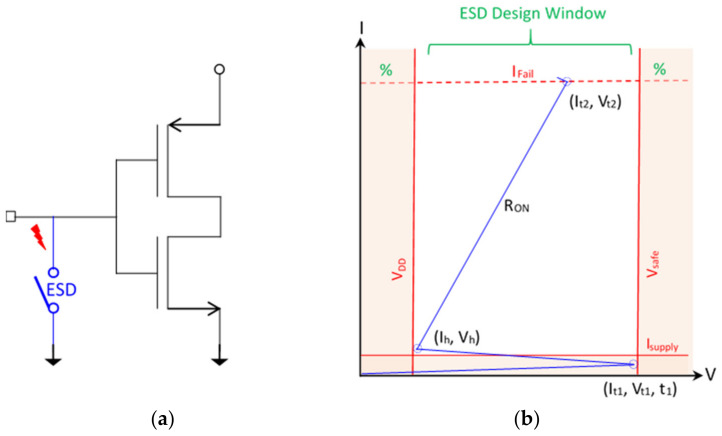
ESD protection concepts: (**a**) an ESD protection device functions as a switch, and (**b**) typical snapback I-V curve of on-chip ESD protection structure featuring ESD-critical parameters that must be designed to comply with an ESD design window.

**Figure 3:**
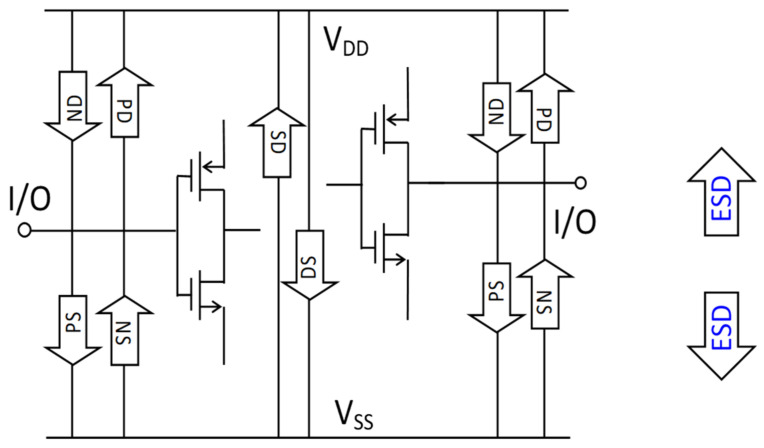
Full-chip ESD protection often requires multiple ESD protection devices per pad to ensure a low-R discharging path between any pairs of bonding pads on a chip. (Arrow boxes representing ESD switch devices in forward low-R conduction mode).

**Figure 4:**
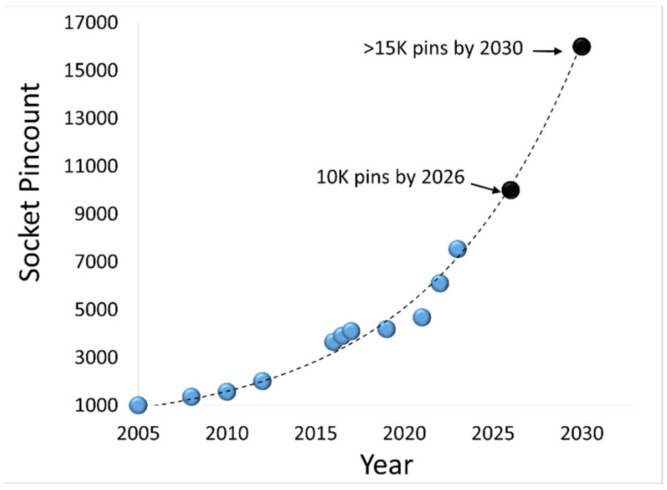
Pad/pin counts for high-performance compute chips increases exponentially over years [21].

Electrostatic discharging occurs when two objects of different electric potentials are brought together, either in direct contact or in close proximity. Electrostatic charges will exchange in between, which produces fast and large current (I) and voltage (V) pulses that can easily damage ICs [14,15]. ESD failure remains a major IC reliability problem to a global microelectronics industry seeking a revenue of ~USD 940 billion by 2030 [16]. On-chip ESD protection is hence required for every IC chip. In principle, an ESD protection structure is placed at a bonding pad on a chip that acts as a controlled switch device, depicted in Figure 2a. The ESD protection switch remains OFF during normal IC operations. When an ESD transient appears at a pad, the ESD switch device will be swiftly turned on to a low-resistance (R_ON_) conduction path to discharge the incident ESD pulse into ground (GND), hence protecting the IC [14]. Figure 2b illustrates a typical snapback I-V characteristic for an ESD protection structure, showing ESD-critical parameters—including triggering voltage, current and time (V_t1_, I_t1_, t_1_), holding voltage and current (V_h_, I_h_,), discharging resistance (R_ON_), and second breakdown voltage and current (V_t2_, I_t2_,)—which must be accurately designed in order to comply with the corresponding ESD design window bounded by the supply voltage (V_DD_), IC breakdown voltage (i.e., safe operation voltage, V_safe_), total supply current (I_supply_) and the maximum sustainable current (I_Fail_), as depicted in Figure 2b [17,18,19]. It is important to know that ESD protection is a full-chip design task. As depicted in Figure 3, full-chip ESD protection ensures that there always exists a low-R discharging path between any two pads on a chip against any incident ESD pulses [19]. Specifically, since an ESD transient may have positive or negative polarity and an ESD switch device is typically one-directional (i.e., optimized for discharging in one direction), multiple ESD protection structures are generally needed per pad to discharge ESD pulses from a pad positively and negatively with respect to positive supply bus (V_DD_) and negative supply lines (V_SS_ or GND), commonly marked as PD/ND, PS/NS, and DS/SD ESD stressing modes, respectively. In addition, ESD design overhead always exists and includes ESD-induced parasitic effects, such as parasitic capacitance (C_ESD_), leakage (I_leak_), and noises, which seriously affect IC performance. In addition, the large size of ESD devices makes whole-chip layout difficult [20]. ESD design overhead becomes a significant design problem for advanced ICs, which often have large numbers of pads, as shown in Figure 4 [21]. Overall, ESD protection design becomes increasingly more involving and challenging for advanced ICs at advanced technology nodes, particularly for HI/chiplet-based SoIC chips using advanced μ-packaging.

### 2.2. Novel Interposer-Based ESD Protection

As discussed previously, the ESD design overhead effect emerges as a major challenge to large/complex chips because the inevitable ESD-induced parasitic parameters can seriously affect IC performance; while large ESD device-head counts not only consume chip area but also complicate chip layout floor planning. The ESD protection design problem becomes much more complicated for SoIC chips due to domain (functional, materials, technology) diversity and interface/boundary (materials, chiplets) complexity. We envision that interposer-based ESD protection will be an important potential solution for smart future SoIC chips in μ-packaging.

**Figure 5:**
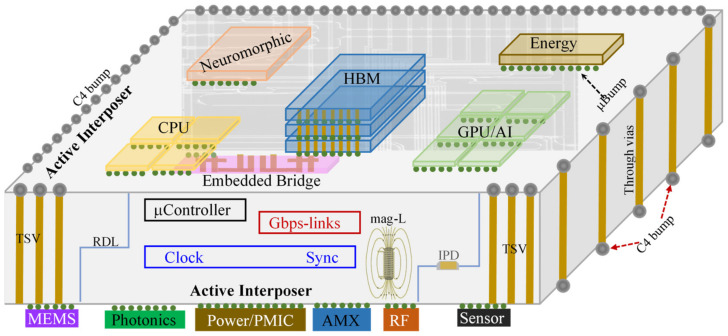
Illustration of a conceptual double-sided active interposer for SoIC packaging (not drawn to scale).

Interposer is a critical technology in 2D/3D ICs and advanced packaging and one that can be used to address the pad/pin pitch mismatch between dies, middle layers and packaging substrate [22]. Typically, a Si interposer serves as an intermediate layer containing complex metal interconnects and through-silicon vias (TSV) to connect chiplets and packaging substrate laterally and vertically, as shown in Figure 1. Interposer technology enables die-level packaging (i.e., IC-scale critical dimensions), supporting heterogeneous integration and terabyte/s high-bandwidth interconnects between dielets. Interposers include Si interposers, organic interposers, glass interposers and embedded interposers (i.e., Si bridges) [23,24,25,26,27]. A passive interposer contains through-vias (e.g., TSV), copper redistributed layer (RDL), Si bridge, and various bonding elements (e.g., micro bumps, copper pillars, Cu–Cu bonding, ball grid arrays, etc.). Recently, active interposers have been developed that contain high-performance integrated passive devices (IPDs) and various supporting circuitry, e.g., clocks, μ-controllers and high-speed links [28,29,30,31]. Figure 5 illustrates a double-sided active interposer for HI/chiplet-based SoIC and μ-packaging with many advanced circuit features.

Clearly, interposers in advanced packaging offer room to host ESD protection structures for both SoC and SoIC chips. In principle, ESD protection devices can be built inside and throughout an interposer, with proper layout planning and alignment so that the ESD switch devices can be connected to pads of chiplets to form a required ESD protection network. One key advantage for interposer-based ESD protection is that the core chip may be “free” of ESD protection devices, or may at least significantly reduce the ESD protection level for a core die (i.e., smaller and fewer ESD devices on a chip), which has been a large ESD design problem in advanced technologies [32,33]. Because of the lack of or reduced ESD protection burden on a chip die, the design of complex ICs in advanced technologies (e.g., very expensive 3 nm CMOS) will focus on delivering high performance, while saving the high-cost die area needed for on-chip ESD protection. Interposer ESD protection will play a more important role in SoICs because the high complexity of 3D heterogeneous chips makes ESD protection a much larger issue in μ-packaging, one for which interposer-based 3D ESD protection will be a vital reliability solution. For large, complex, high-performance chips, the benefits of using interposer-based ESD protection will be more significant because such chips have large numbers of pads (Figure 4); hence, removing/reducing ESD protection in core chips can substantially enhance chip performance while reducing chip costs.

**Figure 6:**
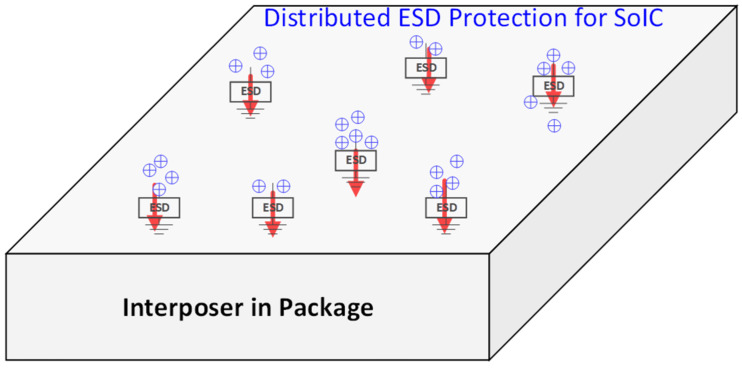
Concept of interposer-based internally distributed CDM ESD protection method.

**Figure 7:**
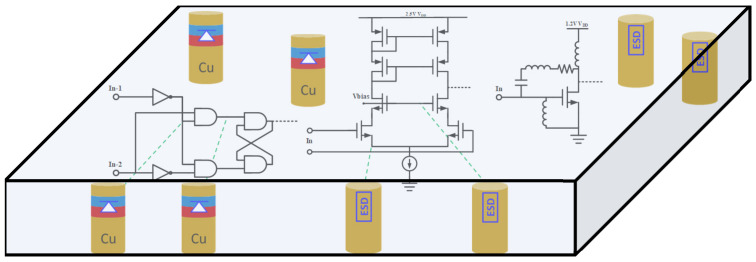
Illustration of internally distributed CDM ESD protection using vertical in-TSV ESD protection devices implemented in either a substrate or an interposer.

More importantly, interposer-based ESD protection is naturally suitable for making a distributed ESD protection network, which is important for charged device model (CDM) ESD protection [34]. Compared with classic human body model ESD protection, CDM ESD is an emerging challenge for modern ICs in advanced technologies [35,36]. With reference to Figure 3, a human body model ESD is a “from-external-to-internal” event, one where the electrostatic charges of a pad originate externally; hence, traditional ESD protection relies on ESD protection structures at the pads (i.e., pad-based ESD protection) to discharge the incident static charges, i.e., preventing external charges from entering into a chip and, therefore, providing ESD protection [14]. On the other hand, CDM ESD is a “from-internal-to-external” phenomenon where static charges induced into a chip over the course of its lifetime are stored and distributed randomly inside an IC die [37]. Due to this unique nature of internal charge distribution, the classic pad-based CDM ESD protection method, in usage for decades, is found to be fundamentally questionable, because when one pad is grounded during CDM ESD stressing, the internal charges must find ways to reach to the GND pad for discharging. Consequently, although at-pad ESD switch devices may conduct properly, the internal routing of ESD currents may still cause internal ESD failures, which is believed to be the main reason for the CDM ESD failure uncertainty that has recently emerged as a significant ESD reliability problem [34]. Clearly, the CDM ESD problem will be much more a design-for-reliability issue for SoICs in μ-packaging. The reason for this is that an SoIC chip has complex internal structures due to the many heterogeneous chiplets and to the packaging structure, making the internal storage of charges within SoICs/packaging much more significant and complicated. As a result, CDM ESD failure will be more uncertain and random, and CDM ESD protection becomes very difficult for SoICs in μ-packaging, something which in turn calls for ESD design innovation. Recently, a novel non-pad-based internally distributed CDM ESD protection method was reported to address this CDM ESD design problem [38]. Instead of using a pad-based CDM ESD protection method, the internally distributed CDM ESD protection method moves ESD switch devices from pads to the internal circuit nodes where static charges are likely stored, as illustrated in Figure 6. Therefore, when internal/local charges accumulate to a given level, the internal CDM ESD switch device will be turned on to discharge the charges locally/internally, without having to route through any complicated internal paths to reach to a GND pad for discharging. Hence, CDM ESD protection can be achieved. Clearly, interposer-based ESD protection offers the unique solution of internally distributed CDM ESD protection for SoICs in μ-packaging, one where local static charges can be readily discharged through ESD switch devices embedded in an interposer, and thus achieving robust CDM ESD protection for SoIC chips. Practically, various ESD switch devices may be used to build an interposer-based ESD protection network, for example, and as shown in Figure 7, an interposer might use vertical in-TSV ESD diodes [39].

## 3. Proof of Concept Design Examples

**Figure 8:**
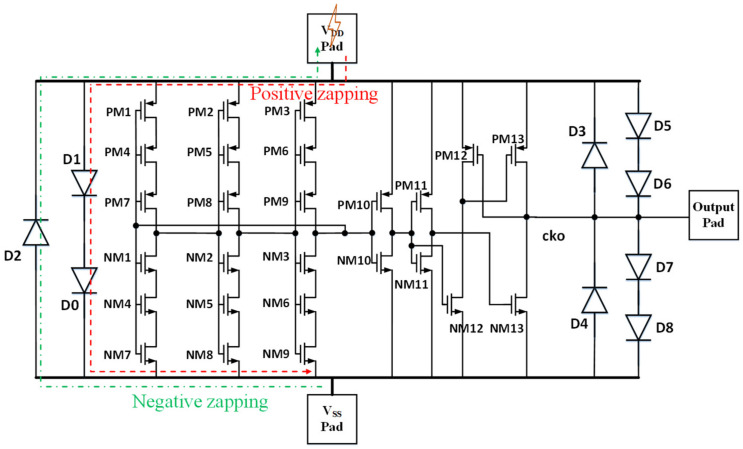
Schematic for a 3-stage oscillator IC featuring a classic pad-based CDM ESD protection network. Under typical CDM ESD zapping (traditional external-to-internal stressing at pad) between V_DD_ pad and V_SS_ pad in both directions, ESD discharging occurs through ESD diodes as designed (dashed-lines indicating ESD discharging paths) [34].

**Figure 9:**
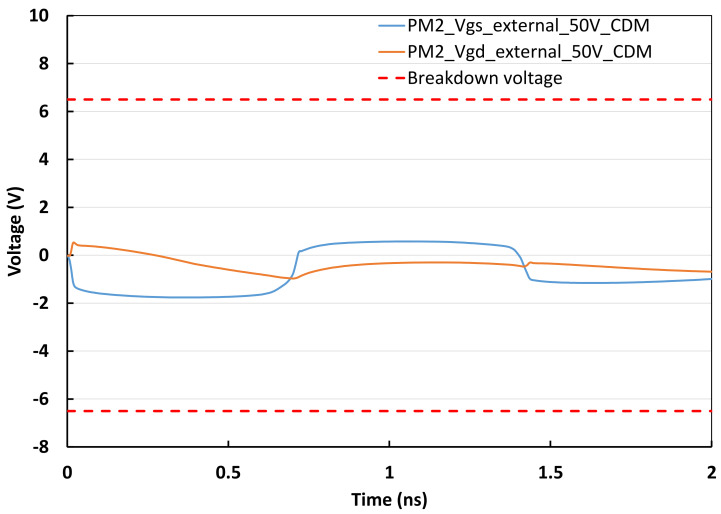
Transient voltage behaviors (V_GS_, V_GD_) of PM2 of the IC in Figure 8 under 50 V CDM ESD stressing (traditional external zapping to pad), suggesting classic pad-based CDM ESD protection seems to be “working” [34].

**Figure 10:**
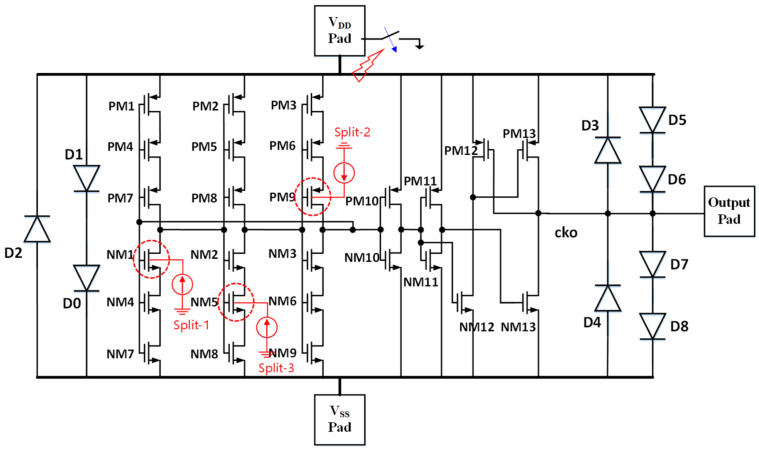
Using the internally oriented CDM ESD zapping (internal-to-external discharging with a pad grounding) method for the same oscillator IC, still using classic pad-based CDM ESD protection. Splits 1, 2 and 3 show three different internal charge storage cases [37].

**Figure 11:**
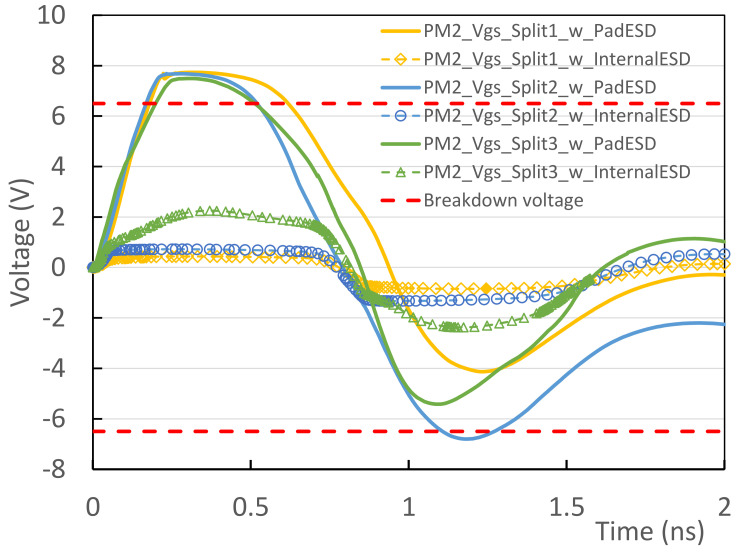
Transient voltage behaviors for typical internal PM2 under internally oriented CDM ESD zapping show that 50 V CDM ESD stressing causes breakdown ESD failure if using classic pad-based CDM ESD protection (solid lines); however, using internally distributed CDM ESD protection (hollow marks) passes 50 V CDM ESD zapping [37].

**Figure 12:**
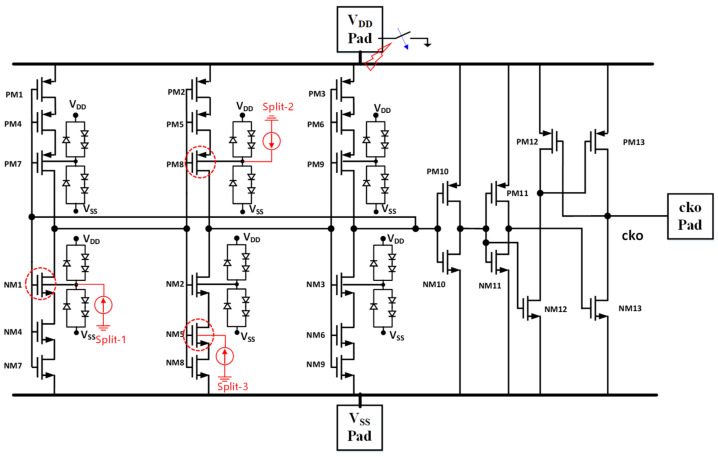
Same oscillator IC uses non-pad internally distributed CDM ESD protection. Applying internally oriented CDM ESD zapping (internal-to-external discharging with one pad grounding). Splits 1, 2, and 3 show three different internal charge storage cases [37].

**Figure 13:**
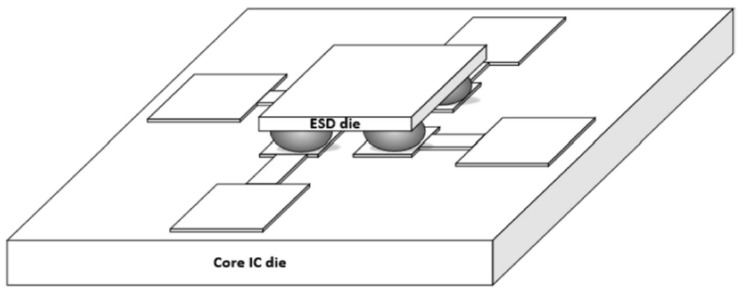
Conceptual illustration of interposer-based ESD protection flip-chip bonded with an ESD-free core die [37].

**Figure 14:**
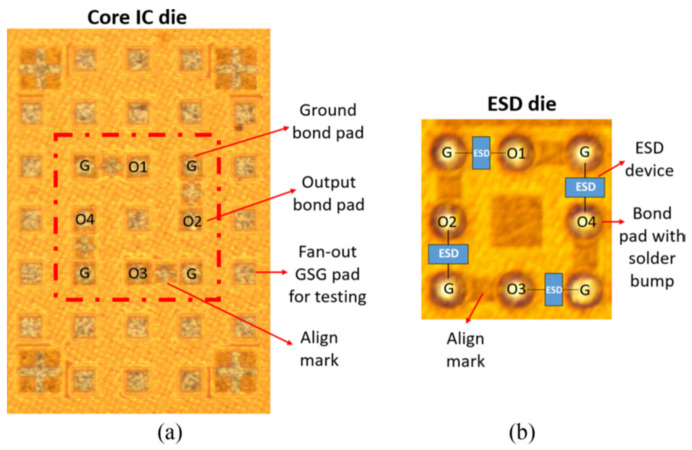
Die photos for an ESD-free SP4T circuit core die (**a**) and a separate interposer ESD die (**b**) [37].

Proof-of-concept prototype designs have been recently reported for interposer ESD protection for single chip [34,37]. A three-stage oscillator IC, designed and fabricated in a foundry with 45 nm SOI CMOS technology, was used as a test circuit. Figure 8 depicts the schematic for the oscillator chip that features classic pad-based CDM ESD protection using diode ESD devices at the pads. ESD simulation shows that, assuming that the ESD pulse comes to the pad externally and under 50 V CDM ESD stressing between V_DD_ pad and V_SS_ pad in both directions, ESD discharging occurs through the ESD diodes at the pads, as designed. Figure 9 presents the transient voltage behaviors of internal transistor voltage, e.g., V_GS_ and V_GD_ of MOSFET PM2, which is much lower than MOSFET breakdown voltage; hence CDM ESD protection seems to work. In accessing ESD protection, gate breakdown voltage is used as one ESD failure criterion, where BV_OX_ ~6.5 V for the 45 nm SOI technology used. Clearly, the traditional “from-external-to-internal” ESD zapping method does not truly reflect the CDM ESD discharging phenomenon, which is a “from-internal-to-external” event. Figure 10 depicts a real CDM ESD zapping scenario following the “from-internal-to-external” approach for the same IC, still featuring pad-based CDM ESD protection. Given that internal charge allocation/storage can be random, the CDM ESD stressing simulation considers three likely charge storage cases—splits 1, 2, and 3 as depicted in Figure 10. Figure 11 shows transient voltage behaviors for the selected internal MOSFET PM2 under the same 50 V CDM ESD zapping (note, discharging originates from internal nodes with one pad grounding). It is readily observed that PM2 fails under the internally oriented 50 V CDM ESD zapping, unlike that shown in Figure 9, hence confirming that classic pad-based CDM ESD protection may not work as expected. Figure 12 depicts the use of non-pad-based internally distributed CDM ESD protection for the same oscillator IC, whose transient voltage behaviors under same 50 V CDM zapping (internal-to-external zapping with one pad grounding) are also given in Figure 11. It is clearly seen that no voltage breakdown occurs, hence the IC successfully passed 50 V CDM ESD zapping. The interposer-based ESD protection concept was also validated experimentally using a single-pole four-throw (SP4T) RF switch circuit and a separate ESD interposer, which can be flip-chip bonded, as depicted in Figure 13 [37]. Figure 14 shows the fabricated SP4T core die (ESD-free) and interposer ESD die fabricated in a foundry 45 nm SOI CMOS technology. It is expected that the same interposer-based ESD protection method should work for SoICs in μ-packaging.

## 4. Interposer-Based ESD Protection Design Challenges

Though very promising, the realization of interposer-based ESD protection for SoICs in μ-packaging will face two major design challenges. First, a new holistic cross-layer/domain ESD protection approach is highly desired for interposer-based ESD protection designs due to the fact that SoICs in μ-packaging involve many, and continuously increasing, chiplets/dielets, different materials and intermediate layers in packaging. Furthermore, many different process technologies are typically used to fabricate different chiplets by different chip vendors. Hence, complying with the ESD design windows (Figure 2b) for all chiplets within one packaging is certainly very difficult. More importantly, holistic ESD protection in packaging will be required to minimize overall ESD design overhead effects that will seriously affect the performance, reliability and costs of SoIC superchips. This is because smart partitioning and allocation of ESD protection switch devices among individual chiplets and packaging media may help to reduce the head counts of all in-packaging ESD protection devices, while still maintaining robust SoIC ESD protection. Relying on local ESD protection on individual chiplets and counting on the simple “stacking” result of SoICs in μ-packaging will not be acceptable. Second, it is well known that a CAD-based design methodology is essential to ESD protection designs for monolithic SoC IC chips in order to achieve ESD protection design optimization, validation and prediction [19]. The same principle applies to holistic ESD protection designs for SoICs in μ-packaging. Due to increased domain/structural complexity, the design and implementation of interposer-based ESD protection for SoICs in μ-packaging will be extremely challenging, such that it could only be tackled by CAD-based design methodologies. Some specific ESD design difficulties include holistic ESD protection co-simulation (atom to system) among all elements in a package; addressing complex interfacing effects across different chiplets and packaging layers and materials; modeling complex boundary conditions and interactions of packaging (e.g., thermal, mechanical effects); including packaging in ESD simulation; new modeling and CAD algorithms and tools to support the above tasks; and new testing techniques to validate ESD protection designs in packaging. All of these are non-trivial R&D tasks for the future. It is also noteworthy that artificial intelligence (AI) techniques will play an important role in developing ESD protection solutions for SoICs in μ-packaging. Particularly, ESD-specific (i.e., scientific) AI models and algorithms should be explored for holistic ESD protection, e.g., modeling internal charge allocation, smart partitioning of SoICs and allocation of ESD protection elements within μ-packaging, to minimize total ESD design overhead effects while achieving high performance and the robust ESD protection of SoICs [17]. We believe that this will be a new ESD protection design area that will attract increasing R&D efforts in the future.

## 5. Summary

Performance and reliability are two core attributes for ICs, including SoICs in advanced packaging. ESD failure is a major IC reliability problem and ESD protection is an emerging design challenge for SoICs in μ-packaging. This article discusses key perspectives for ESD protection research for hetero-integrated SoIC chips, for which interposer-based ESD protection is considered an important potential solution for smart future chips in advanced packaging, one which is articulated using design prototypes. Key design challenges and research directions for exploring holistic ESD protection for SoICs in μ-packaging are highlighted.

## 6. Patents

A. Wang: “Interposer-based ESD Protection Structures”, *U.S. Patent* filed, #62/412,105, 2016.

## Data Availability

Data are contained within the article.

## Abbreviations

The following abbreviations are used in this manuscript:

CDM	Charged device model
CMOS	Complementary metal-oxide-semiconductor
ESD	Electrostatic discharge
HBM	Human body model
HI	Heterogeneous integration
IC	Integrated circuit
SoC	System-on-a-chip
SOI	Silicon-on-insulator
SoIC	Systems-on-integrated-chiplets
